# An Evaluation of the Implementation of the Women, Infants, and Children (WIC) Farmers Market Nutrition Program at Four Chicago WIC Sites

**DOI:** 10.3390/nu15051122

**Published:** 2023-02-23

**Authors:** Jacquelyn Jacobs, Jennifer Holcomb, Helen Margellos-Anast, Linda Martinez, Cindy San Miguel

**Affiliations:** Sinai Urban Health Institute, Sinai Chicago, 1500 South Fairfield Avenue, Chicago, IL 60608, USA

**Keywords:** WIC, food purchases, maternal and child health, food insecurity, farmers’ markets

## Abstract

The Farmers Market Nutrition Program (FMNP), a program of the U.S. Department of Agriculture (USDA), provides coupons to individuals/families enrolled in the Special Supplemental Nutrition program for Women, Infants, and Children (WIC) to purchase fresh produce from approved farmers markets. While some studies suggest FMNP may improve nutrition for WIC clients, there is limited research on program implementation in practice. A mixed-methods equitable evaluation framework was utilized to: (1) better understand the FMNP in practice at four WIC clinics on Chicago’s west and southwest sides that serve predominately Black and Latinx families; (2) detail facilitators and barriers to participation in the FMNP; and (3) describe the potential impact on nutrition. In this manuscript, we present qualitative findings from Aim 1. We identified six steps for how the FMNP has been implemented in our study setting and opportunities to improve the implementation of the program. Findings suggest that well-defined and consistent guidelines on: (1) how to seek state approval for farmers markets; and (2) coupon distribution and redemption, are necessary to optimize utilization. Future research should explore the impact of newly offered electronic coupons on redemption rates and fresh fruit and vegetable purchasing behaviors.

## 1. Introduction

The Farmers Market Nutrition Program (FMNP) (established in 1992) provides coupons to clients of the Special Supplemental Nutrition Program for Women, Infants, and Children (WIC) towards purchasing fruits and vegetables from approved farmers markets, farmers, and roadside stands. As an expansion of regular monthly WIC benefits, FMNP is intended to improve access to fresh fruits and vegetables, reduce food insecurity, and improve nutrition for pregnant and postpartum persons and their families. However, research is inconclusive regarding the extent to which FMNP coupons are redeemed, with some evidence that redemption rates are lower in inner-city communities than they are nationally [1]. Understanding how the FMNP program is currently operating in historically disinvested urban communities is important to better leveraging it and other programs towards addressing the food insecurity that frequently affects such communities.

In 2021, 10.2% of American families reported having experienced low food security (previously known as food insecurity without hunger) at some point over the past 12 months [2]. In the same year, nearly 4% indicated experiencing very low food security (i.e., food insecurity with hunger). The COVID-19 pandemic exacerbated these trends with stay-at-home orders in place and individuals, worldwide, fearing public spaces such as grocery stores. A 2020 Urban Institute report found that nearly 25% of families with school-age children reported household food insecurity in September 2020 [3]. In Chicago, the situation is graver with food insecurity disproportionately affecting families identifying as Latinx or Black (29% and 37%, respectively, vs. 19% overall in 2020) [4]. Another study found that 33% of Chicagoans living in ten predominantly Black and Latinx west and southwest side Chicago communities experienced food insecurity [5]. Food insecurity is associated with poor health outcomes including increased risks of cardiovascular disease, poor mental health, self-control, and memory and learning challenges for children [6,7,8]. Optimizing the uptake of programs such as the FMNP in communities that experience health inequities including disproportionately high rates of food insecurity might be seen as vital to improving health and well-being.

While some studies suggest FMNP coupon redemption can improve the intake of fruits and vegetables by pregnant and postpartum persons, more research is needed to understand the best approaches to improve FMNP coupon redemption rates [9]. Few studies have extended out to assess the impact on a family’s nutrition [1,10,11,12,13,14]. Furthermore, available studies have not examined facilitators and barriers to coupon redemption and increased fruit and vegetable consumption [10,11,12,15], and have had small sample sizes [9,11,15], and relied solely on quantitative methods [1,11,12]. To fill this gap in the research, our study examined the impact of a local partnership between four Chicago-area WIC sites serving some of Chicago’s most economically challenged communities of color, and the Farm on Ogden, an urban greenhouse farmers market, on the access to, purchasing of, and intake of healthy food for low-income pregnant and postpartum persons and their children. The study aims to improve equity in access to healthy foods for low-income pregnant persons and postpartum persons, their children, and their immediate families. We hypothesized that by enhancing our understanding of how FMNP coupons are distributed and utilized, as well as facilitators and barriers to redemption, and the impact of the local FMNP coupon program and partnership with the Farm on Ogden on nutrition, we will be ready to better engage diverse stakeholders and systems to improve food access and nutrition among pregnant and postpartum persons and their families.

## 2. Materials and Methods

### 2.1. Study Design

Via evaluation and research processes that incorporate formative, process, and outcome evaluation methods, along with a mixed-methods design to gauge impact, the study had three specific aims: Aim 1 was to better understand the program in practice; Aim 2 was to examine facilitators and barriers to coupon redemption; Aim 3 was to enhance the impact of a local FMNP program on nutrition. The current manuscript focuses on Aim 1—understanding the program in practice. The Equitable Evaluation Framework and Center for Disease Control and Prevention’s (CDC) Framework for Program Evaluation guided the researchers through each step of the evaluation in order to answer critical questions about the implementation of FMNP and the extent to which it increases fresh fruit and vegetable consumption for pregnant and postpartum persons and their families [16,17]. The Equitable Evaluation Framework is rooted in three principles: (1) In service of and contributes to equity; (2) Can and should answer critical questions about the populations; and (3) Designed and implemented in a way that is commensurate with the values underlying equity work. Following the CDC’s Framework for Program Evaluation, the research team engaged Sinai Urban Health Institute’s (SUHI) Community Advisory Committee made up of individuals (roughly 50% Black and 50% Hispanic/Latinx) who represent the racial diversity of the community, and have a vested interest in improving the health and wellbeing of Chicago communities [18]. The Committee informed study planning and design, recruitment, data collection, and the dissemination of findings. The Mount Sinai Hospital Institutional Review Board approved this project (protocol #20-02). 

### 2.2. Study Setting

Sinai Chicago is Illinois’ largest private safety-net healthcare system, and in addition to clinical care, includes a social-service arm (Sinai Community Institute [SCI]) and a community-engaged research arm (SUHI). Sinai Chicago serves the predominantly non-Hispanic Black and Latinx west and southwest sides of Chicago, areas of the city that have been historically disinvested and are disproportionately impacted by health and social inequities. Chicago is divided into seventy-seven officially recognized community areas [19]. SCI has four affiliated WIC clinics that participated in the study. Three clinics are located within the Chicago city limits in North Lawndale, a predominantly non-Hispanic Black (82.7%) community area on the west side of Chicago, and Chicago Lawn, a primarily Hispanic/Latinx (55.4%) community area on the southwest side [20]. The fourth clinic is in Cicero, a southwest suburb bordering the city that is predominately Hispanic/Latinx (88.9%) [21]. Throughout the COVID-19 pandemic, SCI’s WIC clinics provided high-quality, uninterrupted service to WIC clients and made accommodations in line with WIC waiver flexibilities, such as offering remote counseling appointments to clients, and offering remote benefit issuance. SCI provided participating WIC clients who came to their appointment in person with an FMNP coupon book of four five-dollar coupons, for a total of USD 20. The Farm on Ogden, the farmers market in this study, is an FMNP state-approved urban farm located in Chicago’s North Lawndale community area near the SCI clinics. 

### 2.3. Data Collection and Analysis

A document review, semi-structured interviews, and a mobile survey were conducted to better understand the FMNP in practice. Table 1 includes a full summary of the data collection approach and sources used in the evaluation of this urban FMNP. The three data collection approaches and analyses were completed separately, and findings were triangulated to document processes within the WIC sites, to better understand the distribution of FMNP coupons, and characterize WIC clients and those who received the FMNP coupons.

#### 2.3.1. Document Review

In July 2021, 16 foundational documents related to the Illinois WIC FMNP were received from SCI WIC sites and the Farm on Ogden. All documents were in English. Examples of documents include job descriptions of key staff and an FMNP informational brochure for WIC clients. A full list of documents can be found in the Appendix A, Table A1. A collection matrix was created and used for a content analysis of the documents, focused on identifying program goals, program processes, and key stakeholders. 

#### 2.3.2. Semi-Structured Interviews

Criterion sampling was used to identify interview participants [22]. From July to December 2021, semi-structured interviews were conducted with 11 WIC clients (seven in English and four in Spanish) and 10 SCI-affiliated WIC clinic and farmers market staff. WIC participants were pregnant or postpartum persons aged 18 years and older, spoke English or Spanish, and had received FMNP coupons at one of the SCI-affiliated WIC clinics during the summer/fall 2021 season. Clients were queried on their experiences with the WIC program overall, their experiences receiving and redeeming FMNP coupons, and were asked for recommendations towards the improvement of the local FMNP program. Among staff interviewees, roles ranged from WIC and farmers market leadership (director and manager) to market coordinator, WIC nutrition counselor and nutritionist, and WIC administrative staff. Staff interviews explored decision making around who is offered FMNP coupons, coupon distribution processes, perceptions of facilitators and barriers to redemption, and coupon redemption processes at the farmers market. As a result of the COVID-19 pandemic and surges in cases during the data collection period, all interviews were conducted virtually. Staff interviews were conducted over WebEx and client interviews were conducted over the phone. Interviews were 15 to 45 min in length, audio recorded by one member of the research team, and then transcribed by a professional service. Verbal consent was obtained for clients and written consent was obtained for staff. 

Nvivo (released in March 2020) was used to conduct a thematic analysis of the client and staff interviews. First, four members of the research team reviewed the transcripts for recurrent codes. Two codebooks were developed: (1) a combined codebook for English and Spanish-speaking WIC clients, and (2) a codebook for the staff interviews (SCI-affiliated WIC clinic and urban farmers market). The researchers determined themes and sub-themes using the repetition of words, phrases, and meanings. The researchers then reviewed each other’s themes and discussed discrepancies to arrive at consensus. These methods allowed for a deeper understanding of the themes and relationships that emerged around decision making and regarding who receives FMNP coupons as well as practices across the WIC clinics. Finally, the researchers narrowed the themes and sub-themes based on the most common themes. 

## 3. Results

### 3.1. FMNP in Practice

The document review and semi-structured interviews revealed six key intended steps for the successful implementation of the FMNP. These six key components were examined within the context of the program in practice at the SCI-affiliated WIC clinics serving pregnant and postpartum persons on the southwest and west sides of Chicago. Table 2 summarizes the components of the program, elements in practice, and actors for both. As the first step (Step 1), farmers (i.e., farmers markets and roadside/farm stands) must meet the USDA and state agency requirements for FMNP participation. For those in Illinois, that requires submitting an application to the Illinois Department of Human Services (DHS). Farmers must sign a contract with DHS and receive authorization to accept FMNP coupons in the upcoming season (July through October). WIC managers at local agencies provide training to staff on the FMNP coupons (Step 2). These local agencies generally receive FMNP coupons in July (Step 3), but in the 2021 season, the SCI-affiliated WIC clinics did not receive and distribute these coupons until September. As WIC staff called clients to remind them about their upcoming appointment, they also notified clients that the FMNP coupons were available (Step 4). When clients come in for an in-person appointment, WIC staff conduct an informal assessment to identify if they and/or their family have sufficient food (i.e., “Is there enough food in your household?”). Based on their responses, WIC staff offer the FMNP coupons to clients and provide them with information about eligible food for purchase, coupon increments (i.e., dollar value per coupon), and state-approved farmers market locations for the current season. Clients are informed they must identify an approved location that accepts coupons and redeem the coupons for eligible products. The state-approved locations will accept coupons through the end of October (Step 5). Finally, farmers and/or farmers market staff stamp the coupons with their farmer identification number, and cash or deposit the coupons as checks before the date indicated on the coupon (Step 6).

### 3.2. Selected Staff and Client Interview Themes

#### 3.2.1. Beliefs That FMNP as Intended Is Beneficial

WIC clients, WIC staff, and farmers market staff supported the concept of the FMNP and recognized that the FMNP, in theory, could increase access to fruits and vegetables. Clients who redeemed the FMNP coupons in the current season reported a positive experience at the farmers market and described the importance of redeeming the coupons. Clients were able to supplement family meals with the fruits and vegetables that they were able to buy at the farmers market, as one client mentioned:

*
I was a little short on money…I was trying to find a meal that was suitable for my pocket needs…I found different ingredients in my refrigerator, and there were a few items, like vegetables, that I was missing. And I remembered that I had those coupons…I sent my family member out to go get those vegetables, and it was useful because I didn’t have the total amount, so using the coupons helped me. I had just enough for what I needed [WIC Client 8].
*

WIC staff noted that many clients who were aware of the coupons and had redeemed them in a previous season prioritized the FMNP coupons. Clients asked about the coupons as shared by a WIC staff member:

*
There are participants…since kids can stay in the program until age 5, as long as they’re eligible for WIC, and they meet the criteria of farmers market coupons…that ask us every year… Hey, did the farmer’s market coupons come in? [WIC Staff 4].
*

Clients who had not redeemed the coupons also believed that the farmers markets accepting coupons could provide high-quality, affordable produce compared to their local grocery stores. Some clients had past experiences at farmers markets while others shared that the perception of produce quality was a motivator to redeem the coupons as one WIC client explained:

*
I went to [WIC to] get supplies for my daughter, and when they gave me the coupons, I saw that they were for fresh vegetables and fruit. And, I actually like that because I get to grab fruit that I like, and they’re fresh… [I know] they’re not sitting on the store for a while, not knowing if they’ve gone bad or [if] they’re gonna expire soon [WIC Client 7].
*

Another noted,

*
…I’m hoping that the food is fresher…The place that I was going to…I didn’t get the fruit because I used to get the fruit in the can because the open fruit (produce)… It didn’t look right. So, I’m hoping that it’s fresh fruit…Me and my kids, we eat a lot of fruit [WIC Client 5].
*

#### 3.2.2. Opportunities to Build on Existing Processes and Resources

Clients identified the WIC program as a long-standing, trusted social service program from which they can receive reliable nutrition information and education to support the wellbeing of their family as well as other resources they may not otherwise be able to afford. Clients reported they received information and education about healthy eating, cooking and recipes, breastfeeding, and child development. One client expressed how valuable it is that by participating in a nutrition lesson, she qualified for additional coupons which allowed her to obtain enough milk to last until her next appointment,

*
Something that I didn’t know they was doing; they allow you to do a lesson online and then they give you a month worth of coupons. I’m running out of milk and stuff... So that’s definitely a plus [WIC Client 7].
*

Another client shared how participating in WIC activities allowed her to earn and redeem points for resources to support safe sleep and active daily engagement for her baby.

*
Yeah, [the WIC program] gave us a lot of stuff for my baby, like the points and stuff they was giving me points. And the points build up to get like a playpen, and a bassinet, I have got a lot of stuff from them...toys for my baby [WIC Client 9].
*

Overall, both WIC clients and staff provided positive feedback about the FMNP. However, they also identified potential opportunities to improve program processes and further develop the partnership between communities, organizations, and health centers, which focused on further improvements to accessing fresh fruits and vegetables. Some WIC clients who received FMNP coupons, but did not redeem them, indicated they were unsure if they were provided information about which farmers market locations accepted coupons. As noted by one of those clients:

*
I told [staff member] I didn’t know where [a farmers market] was at and [the staff member] said I could look it up...said I could look it up on the internet [WIC Client 7].
*

To ensure anyone who could benefit from coupons has an opportunity to receive them, one client suggested ensuring that processes are standard across all WIC sites:

*
…just making sure that every counselor is specifically telling every participant like, “Hey, you could go to a farmer’s market,” would definitely be beneficial, and probably improve the program so the people are aware of that benefit [WIC Client 15].
*

Other clients suggested developing partnerships to raise awareness of the FMNP such as organizing smaller pop-up shops in communities where there may not be farmers markets. 

## 4. Discussion

The FMNP is a federally funded program intended to improve access to fruits and vegetables for pregnant and postpartum persons and those with young children in low-income communities. The findings of our study demonstrate the complexity of the real-world implementation of the program in practice on the west and south sides of Chicago and neighboring suburbs. We identified six key components required for implementation of the FMNP: (1) farmers markets seek authorization to accept FMNP coupons; (2) WIC staff receive training on the FMNP program; (3) USDA sends FMNP coupons to participating WIC sites; (4) WIC staff notify clients of FMNP program and assess client nutrition needs to determine coupon issuance; (5) WIC clients identify participating farmers markets and shop for preferred foods; and (6) farmers markets stamp and collect coupons. Having knowledge of how the FMNP operates and documenting these steps has the potential to make the authorization process easier for farmers markets, optimize FMNP coupon distribution within the WIC clinic setting, and ultimately increase access to farmers markets and therefore fresh fruits and vegetables for WIC clients. In order to encourage full participation and maximize benefits for clients, state-specific guidance documenting the program flow should be communicated clearly and accessibly. 

It is worth noting that we found that WIC staff and clients believe the FMNP has the potential to markedly improve access to fresh produce and reduce nutritional inequities. In line with existing research, clients who redeemed their coupons were able to improve the variety and nutritional composition of home-cooked meals with farmers market purchases [13], purchase high-quality produce they may not have found at a grocery store [23], and receive additional nutrition and cooking education [24]. However, we also documented opportunities to improve how the program was communicated to clients. These findings indicate a need for consistent informational material and processes across sites that will better equip clients to utilize coupons.

This study allowed us to fully describe the FMNP in practice from both the perspective of WIC clients who received FMNP coupons, as well as farmers market and WIC staff. We identified six steps for how the FMNP has been implemented in Chicago, as well as opportunities to improve the implementation of the program, including standardizing processes across participating FMNP-distributing WIC clinics and farmers markets, and building upon the trusted relationships many WIC clinics have within their communities. In order to address food insecurity in urban communities such as Chicago, it is important to better understand the programs that exist to reduce these inequities. 

### Strengths and Limitations

A notable strength of this study was our partnership with the community, participating WIC clinics, and an urban farm on the west side of Chicago. Working with these partners allowed us to refine data collection tools, develop recruitment tactics, and interpret our findings within the context of the community of interest. Similarly, our use of equitable evaluation principles to guide this evaluation presents a model for how other studies can partner with stakeholders to conduct evaluations that are grounded in cultural humility and acknowledge historical barriers to equity. Finally, it is important to note that this study was conducted during 2021 when monthly WIC cash-value benefits had increased in response to the COVID-19 public health emergency. As a result, WIC clients were receiving a larger benefit compared to pre-pandemic, but were still interested in additional avenues to access fresh produce.

This study was conducted at the height of the COVID-19 pandemic. As such, there were logistic challenges with the implementation of the program and with data collection. WIC clients were able to meet with their WIC counselors virtually, making it challenging to distribute physical coupons to clients. Similarly, recruitment for our evaluation initially took place in person at WIC clinic sites, and therefore limited research subjects to those who were still coming on site for services. In order to increase our sample, we expanded recruitment methods to using snowball sampling, social media recruitment strategies, and word of mouth with partners. Another limitation was the shortened period for which WIC clients were able to use their coupons. In the 2022 season, participating SCI-WIC clinics did not receive this season’s FMNP coupons for distribution until September. Coupons are only accepted until October, greatly reducing the time period in which clients could utilize them. Finally, our study was not designed to compare outcomes for pregnant versus postpartum persons. Future research should explore the potential barriers that may arise for each of these stages of pregnancy. 

## 5. Conclusions

The present study identified six steps for the real-world implementation of the FMNP in an urban setting. Findings suggest that key stakeholders see significant value in the program, but provided recommendations for improvements in the distribution of coupons, communication about the program, and access to farmers markets. First, there should be clear guidelines and information on the FMNP, including the location of FMNP state-approved farmers markets, hours of the markets, information on available transportation to farmers markets, and product availability. Next, it is important that the coupon season be long enough to provide adequate time for clients to use coupons. Delay in coupon distribution severely limits client ability to purchase foods from farmers markets. It is critical to document clear steps for all actors involved in the program to ensure equitable access and distribution of coupons. States are now able to submit a proposal to the USDA Food and Nutrition Service (FNS) requesting the use of electronic solutions to distribute FMNP coupons instead of using traditional paper coupons [25]. This modernization has the potential to further increase the accessibility of fresh produce and reduce food insecurity but will require formalizing consistent protocols for equitable implementation. Finally, as of February 2023, FNS is accepting comments on proposed changes to the WIC food packages. One of these proposed changes is to make permanent a temporary increase to the amount of fruits and vegetables WIC clients could purchase with their benefits. Findings from the present study support this change, as interview participants indicated substantial interest in improved access to high-quality and affordable fruits and vegetables.

## Figures and Tables

**Table 1:** Data collection in an evaluation of the Farmers Market Nutrition Program (FMNP).

Timeframe	Data Collection Approach	Data Source	Aim
July 2021	Document Review	Illinois WIC FMNP program documents and protocols	Understand process of FMNP coupon distribution *
July–August 2021	Semi-Structured Interviews	SCI-affiliated WIC clinic staff	Understand process of FMNP coupon distribution * Understand the process * and decision-making around who is offered FMNP coupons Examine perceptions of facilitators and barriers to redemption Enhance the impact of a local FMNP program on nutrition
July–November 2021	Semi-Structured Interviews	Urban farmers market staff	Understand how coupon redemption works onsite * Enhance the impact of a local FMNP program on nutrition
September–December 2021	Semi-Structured Interviews	WIC clients who received FMNP coupons	Characterize WIC clients who received FMNP coupons Examine utilization of coupons and experiences accessing the market Examine facilitators and barriers to and perceived benefits of participation Examine underlying structural barriers that impact nutritional disparities Enhance the impact of a local FMNP program on nutrition Assess the impact of the COVID-19 pandemic on WIC clients
	Mobile Survey	WIC clients	Characterize WIC clients Examine differences in fruit and vegetable intake and food attitudes between WIC clients who receive and redeem coupons Assess the impact of the COVID-19 pandemic on WIC clients Enhance the impact of a local FMNP program on nutrition

* Evaluation aims covered in this manuscript.

**Table 2:** Farmers Market Nutrition Program (FMNP) in practice at Sinai Community Institute (SCI)-affiliated WIC clinics on the southwest and west sides of Chicago, 2021 season.

Intended Steps for Implementation	Actors	Program in Practice	Actors
Step 1: Meet U.S. Department of Agriculture (USDA) requirements for FMNP participation	USDA State agency Farmers markets and roadside/farm stands	Complete Farmer Application Form Receive authorization/contract to participate in FMNP	USDA Illinois Department of Human Services (DHS), Bureau of Family Nutrition Urban greenhouse garden and market in Chicago
Step 2: Provide training and monitoring of program	WIC staff	Evaluate WIC staff	WIC Program Manager
Step 3: Receive coupons from state agency in July	USDA State agency WIC local agencies	Receive coupons in September Examine where approved farmers are located compared to SCI WIC clients Distribute coupons to nutritionist at SCI WIC clinics	USDA Illinois DHS Sinai Community Institute (SCI)
Step 5: Redeem coupons by end of October	Eligible WIC Program Clients	Search internet to find approved farmers market or roadside/farm stand	Eligible WIC Program Clients
		Select produce/shop at farmers market Consider family preferences and needs Confirm eligible products to purchase with FMNP coupons Decide on utilization of multiple benefits (e.g., VeggieRx, Supplemental Nutrition Assistance Program (SNAP) /Illinois LINK)	
		Identify family member to redeem coupons Visit farmers market during the weekend	
Step 6: Accept FMNP coupons	Farmers markets and roadside/farm stands	Stamp coupons with farmer ID number as a state-approved FMNP location and send to accounting department	Urban greenhouse garden and market in Chicago

## Data Availability

The data presented in this study are available on request from the corresponding author. The data are not publicly available due to identifying information.

## Appendix A

Document Review List

**Table A1:** Document Review List included in the evaluation of a Women, Infants, and Children (WIC) Farmers Market Nutrition Program (FMNP) in Chicago.

Name of Document	Document Type
Illinois Department of Human Services Farmer Application Letter	FMNP Application Letter
Guide to Illinois Farmers Market Accepting Link, Link Match, and WIC + Senior Coupons, 2019	Informational Brochure from Experimental Station—Link Up Illinois
Illinois WIC and Senior Farmers Market Nutrition Program Brochure	Informational Brochure
WIC/Senior FMNP Farmer Application Form	FMNP Application Form
Illinois Department of Human Services WIC Policy and Procedure Manual	Policies and Procedures
Financial Incentives Increase Purchases of Fruit and Vegetables among Lower-Income Households with Children	Scholarly Article- Moran et al. (2019)
Illinois Department of Human Services FMNP Farmer Training Manual Insert, 2021	Farmer Training Manual Insert
Illinois Department of Human Services FMNP Farmer Review Form, 2020	Farmer Review Form
FMNP Style Guide	Guide
Illinois Farmers’ Market Nutrition Program, 2021-2023	Farmer Training Manual
Windy City Harvest (Farm on Ogden) Market Packet, 2020	Accounting, Produce, and Federal Benefits Log
Sinai Health System WIC Clerk I	Job Description, Performance Appraisal and Competency Evaluation
Sinai Health System WIC Nutritionist I	Job Description, Performance Appraisal and Competency Evaluation
Illinois Department on Aging Planning and Service Areas, May 2021	Map
Illinois WIC FMNP Agencies, 2021	Contact List by County
Examining Barriers to and Facilitators of a Farmers Market Coupon Program Targeting Women and Children in Chicago: An Equity-Focused Evaluation	Sinai Health System Evaluation Grant Proposal for Robert Wood Johnson Foundation

## References

[1] Seidel M., Brink L., Hamilton M., Gordon L. (2018). Increasing WIC Farmers’ Market Nutrition Program Redemption Rates: Results and Policy Recommendations. Prog. Community Health Partnersh. Res. Educ. Action.

[2] Coleman-Jensen A., Rabbitt M.P., Gregory C.A., Singh A. Household Food Security in the United States in 2021. https://www.ers.usda.gov/webdocs/publications/104656/err-309.pdf?v=378.6.

[3] Gupta P., Gonzalez D., Waxman E. Forty Percent of Black and Hispanic Parents of School-Age Children Are Food Insecure. 2020, Urban Institute. https://www.urban.org/research/publication/forty-percent-black-and-hispanic-parents-school-age-children-are-food-insecure.

[4] Food Equity Council Chicago Food Equity Agenda. https://www.chicago.gov/content/dam/city/sites/food-equity/pdfs/City_Food_Equity_Agenda.pdf.

[5] Hunt B.R., Benjamins M.R., Khan S., Hirschtick J.L. (2018). Predictors of Food Insecurity in Selected Chicago Community Areas. J. Nutr. Educ. Behav..

[6] Loftus E.I., Lachaud J., Hwang S.W., Mejia-Lancheros C. (2020). Food insecurity and mental health outcomes among homeless adults: A scoping review. Public Health Nutr..

[7] Liu Y., Eicher-Miller H.A. (2021). Food Insecurity and Cardiovascular Disease Risk. Curr. Atheroscler. Rep..

[8] Grineski S.E., Morales D.X., Collins T.W., Rubio R. (2018). Transitional Dynamics of Household Food Insecurity Impact Children’s Developmental Outcomes. J. Dev. Behav. Pediatr..

[9] Di Noia J., Monica D., Sikorskii A., Cullen K.W. (2017). Outcomes of a randomized controlled trial of nutrition education to promote farmers’ market fruit and vegetable purchases and consumption among women enrolled in the Special Supplemental Nutrition Program for Women, Infants, and Children (WIC). BMC Nutr..

[10] Anderson J.V., Bybee D.I., Brown R.M., McLEAN D.F., Garcia E.M., Breer M., A Schillo B. (2001). 5 A Day Fruit and Vegetable Intervention Improves Consumption in a Low Income Population. J. Am. Diet. Assoc..

[11] Racine E.F., Vaughn A.S., Laditka S.B. (2010). Farmers’ Market Use among African-American Women Participating in the Special Supplemental Nutrition Program for Women, Infants, and Children. J. Am. Diet. Assoc..

[12] Herman D.R., Harrison G.G., Afifi A.A., Jenks E. (2008). Effect of a Targeted Subsidy on Intake of Fruits and Vegetables Among Low-Income Women in the Special Supplemental Nutrition Program for Women, Infants, and Children. Am. J. Public Health.

[13] Savoie-Roskos M., Durward C., Jeweks M., LeBlanc H. (2015). Reducing Food Insecurity and Improving Fruit and Vegetable Intake Among Farmers’ Market Incentive Program Participants. J. Nutr. Educ. Behav..

[14] McCormack L.A., Laska M.N., Larson N.I., Story M. (2010). Review of the Nutritional Implications of Farmers’ Markets and Community Gardens: A Call for Evaluation and Research Efforts. J. Am. Diet. Assoc..

[15] Calo W.A., Marin E., Aumiller B., Murray A., Baptiste C., Bermudez M., Kraschnewski J.L. (2022). Implementing Locally Tailored Strategies to Promote Redemption of Farmers’ Market Nutrition Program Vouchers Among WIC Participants in Central Pennsylvania. Health Promot. Pract..

[16] Equitable Evaluation Initiative Equitable Evaluation Framing Paper. www.equitableeval.org.

[17] Centers for Disease Control and Prevention Framework for Program Evaluation in Public Health. https://www.cdc.gov/evaluation/.

[18] Sinai Urban Health Institute Sinai Community Health Survey 2.0. Community Advisory Committee Members. https://www.sinaisurvey.org/cac-members.

[19] Chicago Data Portal Boundaries—Community Areas. https://data.cityofchicago.org/d/cauq-8yn6?category=Facilities-Geographic-Boundaries&view_name=Boundaries-Community-Areas-current-.

[20] Chicago Metropolitan Agency for Planning Community Data Snapshot—Chicago Lawn. https://www.cmap.illinois.gov/documents/10180/126764/Chicago+Lawn.pdf.

[21] United States Census Bureau Quick Facts—Cicero Town, Illinois. https://www.census.gov/quickfacts/cicerotownillinois.

[22] Palinkas L.A., Horwitz S.M., Green C.A., Wisdom J.P., Duan N., Hoagwood K. (2015). Purposeful Sampling for Qualitative Data Collection and Analysis in Mixed Method Implementation Research. Adm. Policy Ment. Health Ment. Health Serv. Res..

[23] Payne G.H., Wethington H., Olsho L., Jernigan J., Farris R., Walker D.K. (2013). Implementing a Farmers’ Market Incentive Program: Perspectives on the New York City Health Bucks Program. Prev. Chronic Dis..

[24] Dannefer R., Abrami A., Rapoport R., Sriphanlop P., Sacks R., Johns M. (2015). A Mixed-Methods Evaluation of a SNAP-Ed Farmers’ Market–Based Nutrition Education Program. J. Nutr. Educ. Behav..

[25] United States Department of Agriculture WIC FMNP and SFMNP FY 2022 Guidance Package [Press release]. https://www.fns.usda.gov/wic/fmnp-and-sfmnp-fy-2022-guidance-package.

